# Machine learning-based prognostication of mortality in stroke patients

**DOI:** 10.1016/j.heliyon.2024.e28869

**Published:** 2024-04-03

**Authors:** Ahmad A. Abujaber, Ibrahem Albalkhi, Yahia Imam, Abdulqadir Nashwan, Naveed Akhtar, Ibraheem M. Alkhawaldeh

**Affiliations:** a Nursing Department, Hamad Medical Corporation, Doha, Qatar; b College of Medicine, Alfaisal University, Riyadh, Saudi Arabia; c Department of Neuroradiology, Great Ormond Street Hospital NHS Foundation Trust, Great Ormond St, London WC1N 3JH, United Kingdom; d Neurology Section, Neuroscience Institute, Hamad Medical Corporation, Doha, Qatar; e Faculty of Medicine, Mutah University, Al-Karak, Jordan

**Keywords:** Stroke, Prognosis, Mortality, Ischemic stroke, Hemorrhagic stroke, Machine learning

## Abstract

**Objectives:**

Predicting stroke mortality is crucial for personalized care. This study aims to design and evaluate a machine learning model to predict one-year mortality after a stroke.

**Materials and methods:**

Data from the National Multiethnic Stroke Registry was utilized. Eight machine learning (ML) models were trained and evaluated using various metrics. SHapley Additive exPlanations (SHAP) analysis was used to identify the influential predictors.

**Results:**

The final analysis included 9840 patients diagnosed with stroke were included in the study. The XGBoost algorithm exhibited optimal performance with high accuracy (94.5%) and AUC (87.3%). Core predictors encompassed National Institutes of Health Stroke Scale (NIHSS) at admission, age, hospital length of stay, mode of arrival, heart rate, and blood pressure. Increased NIHSS, age, and longer stay correlated with higher mortality. Ambulance arrival and lower diastolic blood pressure and lower body mass index predicted poorer outcomes.

**Conclusions:**

This model's predictive capacity emphasizes the significance of NIHSS, age, hospital stay, arrival mode, heart rate, blood pressure, and BMI in stroke mortality prediction. Specific findings suggest avenues for data quality enhancement, registry expansion, and real-world validation. The study underscores machine learning's potential for early mortality prediction, improving risk assessment, and personalized care. The potential transformation of care delivery through robust ML predictive tools for Stroke outcomes could revolutionize patient care, allowing for personalized plans and improved preventive strategies for stroke patients. However, it is imperative to conduct prospective validation to evaluate its practical clinical effectiveness and ensure its successful adoption across various healthcare environments.

## 1 Introduction

Stroke, a notable global health issue, is recognized as the second most prevalent reason for mortality and a major contributor to persistent impairment [1]. According to the World Health Organization, around 13.7 million individuals have a stroke each year, leading to approximately 5.5 million fatalities due to its associated complexities [1]. Furthermore, stroke stands as a key factor in long-lasting disability, with more than 50% of individuals aged 65 and older who survive a stroke encountering diminished physical mobility [2,3].

Qatar, a prosperous peninsula located on the northeastern border of the Arabian Peninsula, has a native Qatari population that constitutes just 15% of the total populace [4]. Despite its wealth, the country grapples with notable public health issues including a high prevalence of obesity, diabetes mellitus (DM), and cardiovascular disease [5]. As of 2020, Qatar held the 15th spot worldwide for obesity, affecting over 35% of its citizens. Moreover, in 2013, approximately 16% of the population were diagnosed with diabetes mellitus [6]. Despite these worrisome figures, Qatar maintains a comparably low stroke incidence rate of 58 cases per 100,000 individuals, significantly undercutting the MENA region's rate of 250 cases per 100,000 people [7,8]. Furthermore, Qatar experiences a relatively low rate of stroke-related fatalities [7].

This can be attributed to the distinct demographic composition where the expatriate population of working age forms the majority [4,9]. The population displays an average stroke occurrence rate of 92.04 per 100,000 adults. The typical age for experiencing a stroke is about 64 years, with the initial cerebrovascular incident commonly happening around 63 years of age. Among the various stroke types, ischemic stroke (IS) prevails, accounting for 73.7% of cases, primarily caused by small vessel disease. Hypertension and diabetes hold particularly high prevalence within this group, impacting 82.7% and 71.6% of patients, respectively. In comparison to males, Qatari females tend to face stroke at older ages, encounter higher rates of hypertension and diabetes, and exhibit an elevated likelihood of disability or death within 90 days following the stroke event [10,11].

Several predictive models exist to anticipate stroke mortality. The Acute Physiology and Chronic Health Evaluation II (APACHE II) is popular among intensivists, while others employ the Sequential Organ Failure Assessment (SOFA). Both tools have demonstrated effective predictive capability, as indicated by accuracy and the area under the curve (AUC) [[12], [13], [14]]. Noteworthy, previous studies have employed logistic regression-based models to predict stroke outcomes. A systematic review, examining the efficacy of different stroke prognostic models based on logistic regression, found the overall predictive performance to be satisfactory, with a median AUC of 0.8. This highlights the need for more robust prognostic models [15].

Over the past decade, there has been a substantial rise in the adoption of machine learning algorithms to forecast stroke outcomes [16]. These algorithms have demonstrated comparable, if not better, capabilities in foreseeing short, medium, and long-term stroke prognosis. In numerous cases, their accuracy and AUC surpassed 0.9 [[17], [18], [19]]. The remarkable performance and potent predictive abilities of machine learning have paved the way for tailoring healthcare to align with the unique care requirements of individual patients. As a result, the healthcare sector is rapidly embracing the utilization and implementation of machine learning models [16,20].

Previous research has identified several factors that influence stroke outcomes. These variables encompass patient demographics, clinical attributes, treatment approaches, and the application of machine learning models for prognostic purposes, including short and long-term mortality [21]. Importantly, elements like age, sex, and the health status before the stroke event, often assessed using the pre-admission modified Rankin Score (mRS), have been recognized as pivotal influencers on patients' prognosis following thrombectomy [22]. Furthermore, lifestyle behaviors, such as smoking, have been identified as having an impact on stroke outcomes, with non-smokers exhibiting a greater likelihood of achieving a favorable recovery [23].

Clinical indicators are important factors in predicting stroke outcomes. For instance, the extent of infarction has been linked to clinical consequences following an IS. Smaller infarct expansion and favorable initial perfusion have been associated with better patient results [24]. Similarly, post-thrombectomy, the National Institutes of Health Stroke Scale (NIHSS) scores and the necessity for decompressive hemicraniectomy have emerged as crucial predictors of mortality [22,25]. Furthermore, certain studies have identified the body mass index's (BMI) relevance in predicting mortality among stroke patients [26]. Hospital-acquired infections, such as urinary tract infections and pneumonia, have been noted as significant factors in forecasting stroke prognosis, and mortality [12,27]. In addition to clinical findings and stroke severity upon presentation, patients' comorbid conditions, such as diabetes mellitus (DM), hypertension (HTN), dyslipidemia, prior stroke, coronary artery disease (CAD), history of atrial fibrillation (AF), or congestive heart failure (CHF), have demonstrated substantial predictive roles in shaping stroke patients' prognosis, including disabilities and mortality [21,22,25,28]. Further factors, such as decrease in the duration from stroke onset to hospital arrival, prompt intervention, especially thrombolysis for IS, increases the chances of a favorable prognosis [[29], [30], [31]].

Anticipating stroke-related mortality holds substantial significance, aiding clinicians in tailoring care plans and enabling patients and their loved ones to foster practical anticipations [32]. Hence, this research endeavor seeks to formulate and assess a machine learning-driven model aiming to predict the one-year mortality rates for both IS and hemorrhagic stroke (ICH) at the onset of the event.

## 2 Material and methods

### Ethical approval

The research obtained authorization from the institutional research board (IRB) at Hamad Medical Corporation, Qatar, under reference MRC-01-22-594.

### 2.1 Data collection

Data were gathered from the Stroke Registry at Hamad General Hospital (HGH), covering the period from January 2014 to July 2022. The dataset encompasses all individuals aged 18 years and older, who were admitted to HGH with a principal diagnosis of stroke. This encompasses cases of IS, transient ischemic attack (TIA), ICH, and stroke mimic. A grand total of 15,859 patients have sought specialized stroke treatment at the hospital since the inception of the stroke registry in Qatar and been previously described [33].

### 2.2 Baseline variables

The gathered variables encompassed various facets of patients' profiles, including demographic information, ethnicity, hemodynamic measurements upon admission (such as heart rate (HR) and blood pressure (BP)), factors contributing to stroke risk, known coexisting conditions, admission location, outcomes during hospitalization (e.g., length of stay (LOS) and occurrences of hospital-acquired infections like pneumonia and urinary tract infections), mortality, and the severity of the stroke event. Stroke severity at admission was calculated utilizing the National Institute of Health Stroke Score (NIHSS) [34,35]. Upon admission, the modified Rankin Scale (mRS) was recorded, representing the patient's condition before the stroke incident, rated on a scale from 0 to 6 [17]. The IS's underlying cause was determined through the Trial of Org 10172 in Acute Stroke Treatment (TOAST) classification [36]. A stroke type variable was generated by amalgamating the five TOAST categories under IS, while intracerebral hemorrhage (ICH) constituted a separate category, facilitating a comparative analysis between these two stroke types. To define BMI categories, the CDC's five-tier definition for adult overweight and obesity was adopted [37].

In terms of ethnicity, patients were categorized into five distinct groups based on their declared nationality: Qatari, Middle East and North Africa (MENA) region, South Asia region, South East Asia region (defined according to the United Nations geo-scheme), and all other nationalities grouped under "other" [[38], [39], [40]]. Noteworthy is the separate classification for Qatari patients, undertaken to facilitate a meaningful comparison. This consideration acknowledges the distinct demographic composition of the country, where expatriates form a substantial part of the population [4,9]. This methodology has been consistently employed in previous research that studies stroke in Qatar [38,41]. All pertinent risk factors, including existing health conditions and smoking history, were ascertained during the patient's hospitalization, and verified by stroke registry personnel through accessing electronic health records. The data is presented in Table 1.

**Table 1:** Statistical characteristics of the collected stroke dataset.

Variable	Feature	Alive	Deceased	Total
Age (year)	<mean (54.1)	5103	211	5314
	≥mean (54.1)	4226	300	4526
	Mean ± SD (54.1 ± 13.5)-IQR 18			
Sex	1: Male	7576	358	7925
	2: Female	1762	153	1915
Ethnicity	1: Qatari	1528	133	1661
	2: MENA	1627	103	1730
	3: South Asian	4865	204	5069
	4: South-East Asian	901	51	952
	5: Other	408	20	428
Mode of Arrival	1: Ambulance	6934	416	7350
	2: Private vehicle	2250	50	2300
	3: In-hospital	145	45	190
Onset of symptoms to hospital arrival	1: ≤3 h	2839	218	3057
	2: 3–6 h	1167	52	1219
	3: 6–24 h	1622	49	1671
	4: >24 h	3005	77	3082
	5: Undetermined	696	115	811
Heart rate at admission (bpm)	<mean (82.9)	4896	232	5128
	≥mean (82.9)	4293	266	4559
	Mean ± SD (82.9 ± 15.4)-IQR 20			
Systolic blood pressure at admission (mmHg)	<mean (160.1)	5152	296	5448
	≥mean (160.1)	4142	210	4352
	Mean ± SD (160 ± 32.9)-IQR 44			
Diastolic blood pressure at admission (mmHg)	<mean (93.1)	5165	303	5468
	≥mean (93.1)	4121	202	4323
	Mean ± SD (93.1 ± 21.1)-IQR 26			
Modified Rankin Score (mRS) pre-stroke onset	<mean (0.4)	8199	332	8531
	≥mean (0.4)	1130	179	1309
	Mean ± SD (0.4 ± 1.1)-IQR 0			
NIHSS at admission	<mean (6.4)	6611	99	6710
	≥mean (6.4)	2718	412	3130
	Mean ± SD (6.4 ± 6.9)-IQR 6			
Body Mass Index (BMI)	1: Underweight	436	69	505
	2: Normal weight	2778	145	2923
	3: Overweight	3778	186	3964
	4: Obese	1613	75	1688
	5: Extremely Obese	724	36	760
Diabetes Mellitus (DM)	0: No	4443	255	4698
	1: Yes	4886	256	5142
Hypertension (HTN)	0: No	2471	157	2628
	1: Yes	6858	354	7212
Dyslipidemia	0: No	5271	371	5642
	1: Yes	4058	140	4198
Prior stroke	0: No	8309	445	8754
	1: Yes	1020	66	1086
Atrial Fibrillation (AF)	0: No	8909	434	9343
	1: Yes	420	77	497
Coronary Artery Disease (CAD)	0: No	8316	418	8734
	1: Yes	1013	93	1106
Tobacco use	0: No	7251	474	7725
	1: Yes	2078	37	2115
Hospital Length of Stay- LOS (days)	< mean (6.56)	6839	277	7116
	≥ mean (6.56)	2490	234	2724
	Mean ± SD (6.5 ± 8.6)-IQR 4.5			
Admission location	1: Stroke Unit	4458	86	4544
	2: ICU	920	239	1159
	3: Other	3951	186	4137
Hospital acquired Pneumonia	0: No	8866	392	9258
	1: Yes	463	119	582
Hospital acquired Urinary Tract Infection (UTI)	0: No	9005	472	9477
	1: Yes	324	39	363
Stroke type	1: Ischemic Stroke (IS)	7881	302	8183
	2: Hemorrhagic Stroke (ICH)	1448	209	1657
One year Mortality	0: Alive 1: Deceased	9329	511	9840

### 2.3 Outcome variable

Mortality at one year was the outcome of interest.

### 2.4 Inclusion/exclusion criteria

This study included all adult patients (aged ≥18) who were diagnosed with IS or ICH. Out of the initial pool of 15,859 patients, 9,840 adults were determined to have IS or ICH, while 6,019 cases encompassing TIA and mimics conditions were excluded from the analysis.

### 2.5 Handling missing data and class imbalance

In instances where data value was missing, we adopted the Multiple Imputation using Chained Equations (MICE) technique to generate data imputations [42]. Within the dataset, it was observed that the HR exhibited the highest proportion of missing data at 1.5%, followed by diastolic blood pressure (DBP) at 0.5%, and systolic blood pressure (SBP) at 0.4%. The cohort had a mortality rate of 5.2%, leading to a concern regarding class imbalance (dead vs alive). To address this issue, diverse strategies were employed based on different machine learning models. For methods such as XGBoost and Random Forest (RF), we integrated class weighting to counteract the imbalance [43,44]. Specifically, we assigned class weights inversely proportional to class frequencies, granting greater weight to the minority class (patients who died) to enhance their impact during the training process.

With regards to the remaining machine learning models, such as Support Vector Machine (SVM), K-Nearest Neighbors (kNN), Random Forest (RF), Logistic Regression (LR), and Naive Bayes (NB), we employed random under-sampling (RUS) [[45], [46], [47], [48], [49]]. Prior to training each model, RUS was applied to the majority class, creating a dataset with balanced proportions. Through this re-sampling approach, instances were randomly removed from the majority class to align with the quantity in the minority class. This strategic alteration effectively countered the issue of class imbalance for these specific models.

### 2.6 Model training and evaluation

The dataset was allocated into a training set (80%) and a validation set (20%) through stratified random sampling. Our models were constructed using the training dataset, and their effectiveness was gauged using the validation dataset. Eight machine learning models were trained, which encompassed weight adjusted XGBoost and RF, as well as SVM, NB, kNN, LR, and RF combined with random under-sampling of the majority class.

Multiple classification metrics were utilized to assess the efficiency of the models, namely accuracy, precision, specificity, recall, F1-score, area under the receiver operating characteristic curve (AUC), Matthew's correlation coefficient (MCC), log loss, and Brier score [[50], [51], [52], [53], [54]]. These metrics provide insights into the model's aptitude for accurately classifying both positive instances (patients who deceased) and negative instances (patients who survived), while factoring in the class imbalance. The model exhibiting the highest F1-score will be chosen as the primary model for subsequent external and temporal validation.

SHAP (SHapley Additive exPlanations) is a powerful library used for explaining the predictions of machine learning models [55]. This tool generates individual-level feature importance scores, referred to as SHAP values. These values quantify the contribution of each feature to a particular prediction outcome.

## 3 Results

**Fig. 1:**
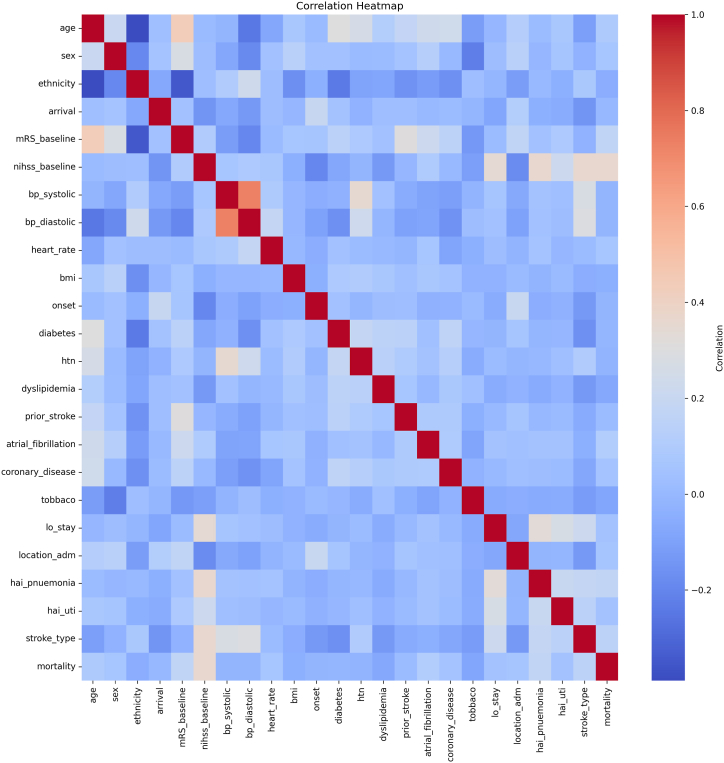
Correlation heat map.

Table 1 outlines the attributes of the study population, with an average age of 54.1 ± 13.5 years. Around 80% of the participants are male, and a mean NIHSS of 6.4 ± 6.9. Approximately 83% of patients had IS diagnosis, with an average length of hospital stay at 6.5 ± 8.6 days. Among the 9,840 patients, 511(5.2%) had died within a year following their stroke. As depicted in Fig. 1, the correlation heat map highlights a significant correlation between the NIHSS upon admission and subsequent mortality.

## 4 Evaluation of trained models’ performance

The XGBoost classifier displayed remarkable accuracy at 94.5%, accompanied by moderate precision (47.8%) and a significant AUC (87.3%). The weight-adjusted Random Forest excelled further, achieving an accuracy of 95.4% coupled with high precision (81.0%) and specificity (99.8%), along with a robust AUC (90.7%). The Random Forest model with random under-sampling demonstrated an accuracy of 81.7% and notable recall (81.7%). Support Vector Classifier (SVC) and Logistic Regression produced accuracies of 84.4% and 82.9%, respectively, with SVC displaying superior recall (81.7%). Gaussian Naive Bayes garnered an accuracy of 80.1%, while the K-Neighbors Classifier attained a score of 78.9%.

**Fig. 2:**
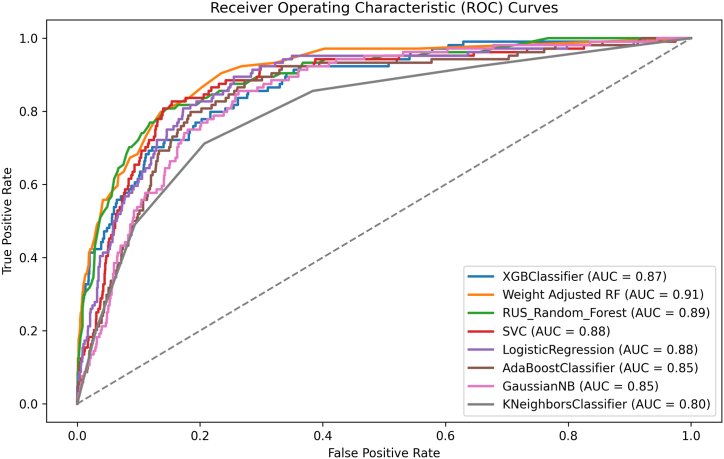
Area Under the Curve for the trained models.

The initial evaluation methods highlighted that the weight-adjusted RF model displayed the most favorable predictive performance, while the KNN model exhibited the least promising predictive capability. Given the presence of class imbalance within our dataset, we employed the F1-score and MCC as criteria for model selection. The outcomes consistently indicated XGBoost classifiers (F1 = 0.443, MCC = 0.416) as the model with the best performance. Calibration assessment of our models was conducted using the Brier score, with the XGBoost classifier demonstrating the most desirable calibration (Brier score = 0.047), while the Adaboost classifier presented the least favorable calibration (Brier score = 0.243). Additionally, considering the log loss metric, the XGBoost classifier displayed a log loss of 0.194, indicating its good prediction ability. As a conclusion of our investigation, the XGBoost classifier was selected as the primary model for forthcoming external and temporal validation, as outlined in Table 2 and Fig. 2.

**Table 2:** Machine learning models performance.

Model	Accuracy	Precision	Specificity	Recall	F1-Score	AUC-ROC	MCC	Log Loss	Brier Score
XGB Classifier	0.945	0.478	0.975	0.413	0.443	0.873	0.416	0.194	0.047
Weight Adjusted RF	0.954	0.810	0.998	0.163	0.272	0.907	0.351	0.178	0.037
RUS Random Forest	0.817	0.199	0.817	0.817	0.320	0.894	0.344	0.391	0.125
SVC	0.848	0.232	0.849	0.817	0.362	0.876	0.383	0.447	0.131
Logistic Regression	0.829	0.206	0.832	0.779	0.325	0.876	0.342	0.448	0.135
AdaBoost Classifier	0.809	0.190	0.810	0.798	0.307	0.850	0.327	0.680	0.243
Gaussian NB	0.801	0.176	0.804	0.750	0.285	0.849	0.297	1.784	0.185
K-neighbors Classifier	0.789	0.161	0.793	0.712	0.262	0.805	0.267	1.550	0.155

AUC area under the curve, ROC receiver operator curve, MCC Matthews correlation coefficient, XGB extreme gradient boosting, RF random forest, RUS random under-sampling, SVC support vector classifier, NB naïve bayes.

### 4.1 SHAP analysis

**Fig. 3:**
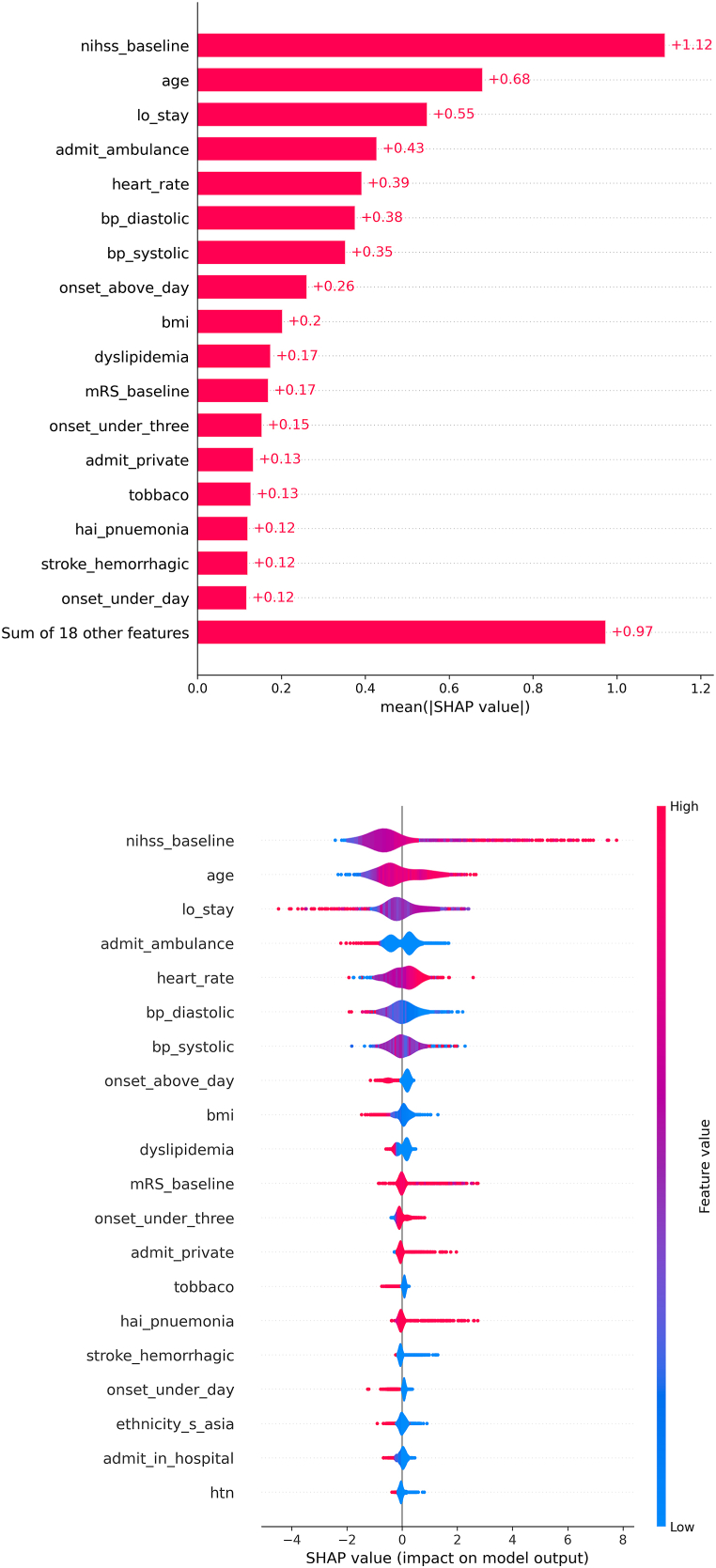
a: SHAP analysis. b: SHAP analysis.

For SHAP analysis, the XGBoost model was chosen to reveal the global and localized distribution of the influence of each input on the model's behavior. The outcomes showed that the baseline NIHSS had the most substantial impact on the model's output, followed by age, LOS, and mode of arrival (Fig. 3 a&b). In another word, Elevated NIHSS and age were linked to stroke mortality. Conversely, mortality was associated with not arriving by ambulance and lower DBP.

## 5 Discussion

This study investigated the efficacy of eight machine learning models and leveraged SHAP analysis to identify the core predictors for stroke mortality. The findings underscored the superiority of the XGBoost model over other models. SHAP analysis conducted on the XGBoost model brought to light the key variables enhancing its predictive ability. These included the stroke's severity (i.e., NIHSS upon admission), age, hospital LOS, mode of arrival, the patient's hemodynamic status represented by HR and BP at admission, symptom onset, and the patient's BMI. Notably, the remaining variables exhibited low SHAP values, suggesting their minimal contribution to the model's predictive output. Importantly, barring the LOS, all other influential factors can serve as early indicators of mortality as they can be assessed upon admission. Consequently, this research might enhance clinicians' ability to forecast mortality promptly and tailor care strategies that address mortality risk [56]. Moreover, it could aid families in forming realistic anticipations regarding expected outcomes, thus enhancing their involvement in the care delivery plan [57].

In line with previous research findings, the NIHSS emerged as the most influential factor in mortality prediction [21,58]. The average NIHSS in our study stood at 6.4 ± 6.9. It was noted that the mean NIHSS for deceased patients significantly exceeded that of survivors (17.14 vs. 5.8), p-value <0.05.

As seen in prior literature, there is a well-established correlation between higher age and increased stroke mortality [26,58,59]. In this study, the mean age was 54 ± 13.5 years, which could be considered comparatively lower when assessed on regional and global scales [7]. The supplementary analysis showcased that 6% of patients surpassing the mean age deceased, in contrast to 4% of others (p-value <0.05). Notably, patients who died exhibited a significantly higher mean age compared to those who survived (59.5 vs. 53.8) with a p-value <0.05.

The LOS contributes to predicting stroke outcomes [60,61]. This study showed that LOS significantly enhances the predictive capability of the model (Fig. 3 a&b). Typically, a lengthier LOS is linked to in-hospital complications, such as hospital-acquired infections, which can potentially lead to mortality [62]. The mean duration of LOS was 6.45 ± 8.6 days. Notably, 8.6% of patients with a LOS exceeding the mean value encountered mortality, in contrast to 3.9% of those with a shorter LOS (p-value <0.05). Furthermore, it was observed that the mean LOS for patients in the mortality group exceeded that of the group who survived (8.5 vs. 6.5 days) with a p-value <0.05. In providing a more contextual understanding, it is imperative to emphasize that LOS can be viewed as a function of various factors, including stroke severity. This suggests that the association between stroke mortality and LOS is variable [63,64]. This study additionally found that patients who exceeded the mean LOS (6.45 days) exhibited significantly higher NIHSS compared to the other group (11 vs. 4.61 days) with a p-value <0.05, aligning with previous literature.

The mode of arrival at the emergency department post-stroke onset plays a significant role in predicting stroke outcomes [65,66]. This can be related to several factors. For instance, as demonstrated by Mohammad YM (2008)., patients arriving via ambulance tend to receive a higher level of and faster care compared to those arriving via private vehicles or walk-in, potentially leading to improved outcomes [65]. The decision to opt for ambulance service or private vehicles/walk-in is often influenced by various factors, including the perceived severity of the illness [67]. Family members might interpret symptoms as mild and choose non-ambulance transportation means. In the present study, it was revealed that patients arriving via ambulance to the emergency department exhibited higher stroke severity compared to those employing alternative modes of transportation (7.2 vs. 3.5) with a p-value <0.05. However, previous reports have indicated that patients experiencing in-hospital stroke have higher mortality rates in contrast to those experiencing out-of-hospital stroke [68], a finding aligned with our study. Specifically, it was identified that 23% of patients encountering stroke during hospitalization had mortality, compared to 5.7% and 2.2% for those developing stroke outside the hospital and arriving via ambulance or other means, respectively. This observation can also be linked to stroke severity, with those undergoing in-hospital stroke exhibiting significantly higher NIHSS compared to those arriving via ambulance (10.93 vs. 7.2) with a p-value <0.05.

Numerous researchers have explored the connection between initial vital signs, particularly HR and BP, and disease outcomes [69,70]. Hebun and colleagues highlighted that admission HR can stand as an independent and potent predictor for acute IS mortality [71]. In this study, the mean HR stood at 83 ± 15.4. The supplementary analysis revealed that deceased patients exhibited a higher average HR upon emergency department arrival compared to the group that survived (87.3 vs. 82.6), p-value <0.05. Furthermore, individuals presenting to the ED with tachycardia (>100 bpm) displayed notably higher NIHSS scores and increased mortality rates relative to those without tachycardia (8 vs. 6) and (9.7% vs. 4.5%) respectively, p-value <0.05. This suggests that HR could potentially be targeted therapeutically to enhance stroke outcomes.

Likewise, the BP recorded upon admission to the emergency department has emerged as a predictive factor for stroke mortality [69,72,73]. While certain previous studies suggest that SBP can independently predict stroke mortality, with DBP holding less significance [69], other researchers have indicated that DBP plays a more notable role, particularly in specific contexts such as in hypertensive patients [73], or even acts as an independent predictor [74]. In the present investigation, it was observed that the admission BP enhances the predictive capacity of the model. Interestingly, a SHAP analysis underscored that DBP yields a higher SHAP value than SBP (Fig. 3 a&b). The visual depiction clearly highlights that lower DBP readings wield a stronger influence on mortality prediction, implying a stronger association between lower DBP and mortality. Upon secondary analysis, the average DBP was measured at 93 ± 21 mmHg. Notably, the average DBP for the studied group was 93 ± 21 mmHg. The group of individuals who died had significantly lower average DBP values compared to those who survived (91 vs. 93 mmHg), p-value <0.05. Moreover, the proportion of patients who died and had DBP readings below the average DBP of the study was higher compared to those who died with DBP readings above the average (5.5% vs. 4.7%), p-value <0.05. On the other hand, there was no significant difference in average systolic blood pressure (SBP) between the group of patients who died and the group that survived (p-value >0.05).

The duration between the onset of stroke and hospital arrival proves influential in predicting stroke outcomes [75], as it determines the speed of care delivery, encompassing interventions like thrombolysis/thrombectomy for IS cases and neurosurgical procedures, all contributing to improved prognostic outcomes [65,76,77]. The SHAP analysis underscores the substantial contribution of patients who present to the hospital beyond a day from the onset to the predictive performance (Fig. 3 a&b). Further analysis pinpoints that arriving at the emergency department within the initial 3 h from onset is correlated with a higher mortality rate compared to arrivals within 3–6 h, 6–24 h, and over 24 h (7.1% vs. 4.3%, 2.9%, and 2.5% respectively, (p-value <0.05). Notably, when contrasting patients presenting within 3 h with those whose onset-to-arrival time is indeterminate, the latter group demonstrated a greater proportion of mortality: 7.1% vs. 14.2% (p-value <0.05). This finding diverges from the common literature results [75]. To offer context, it is crucial to emphasize that much literature concentrates on forecasting mortality in IS, rarely presenting a comparative viewpoint between the outcomes of ICH versus IS. Additionally, in this secondary analysis, it was identified that most patients presenting earlier to the hospital were diagnosed with ICH (28.2% vs. 12.3%, 8.5%, 8.1%, and 31.1% for the other groups respectively). This is linked with significantly more severe stroke presentations, evident in the "<3 h" group with a mean NIHSS of 9 compared to 6.3, 5.4, and 3.4 for the other groups respectively (p-value <0.05). Likely, this outcome can be attributed to the fact that a more severe presentation compels patients' families/significant others to seek the quickest mode of transportation to the hospital. This is substantiated by the discovery that 86.3% of patients arriving within 3 h from onset arrived via ambulance, in contrast to an average of 72% (p-value <0.05).

SHAP analysis corroborated that BMI significantly contributes to the model's predictions. In general, obesity stands as a notable risk factor for stroke development and poor prognosis [21,26]. However, a considerable body of prior research has shown a counterintuitive correlation between BMI and stroke outcomes, reflecting a negative association [21,[78], [79], [80]]. This study similarly discovered that individuals with a BMI <18 (classified as underweight) exhibit a higher mortality rate compared to the mean of the other BMI categories (13.7% vs. an average of 4.7% for remaining BMI categories, p-value <0.05). This phenomenon is recognized as the "obesity paradox" within stroke, where obese patients display better prognoses than those of normal weight or underweight [79,81]. This paradox extends beyond stroke, manifesting in conditions like end-stage renal disease [82]. The underlying reason behind this paradox is attributed to inherent limitations in BMI measurement, as it fails to distinguish between fat, muscle, and skeletal weight, which could play a protective role against certain adverse outcomes [83]. Recent reviews have revealed that methodological robustness influences this phenomenon, stemming from BMI's inaccuracy in measuring obesity, misestimation of body weight, inadequate adjustment for comorbidities, non-linear BMI-outcome relationships, short follow-up periods, and potential selection bias due to the retrospective nature of much prior literature [80,81].

## 6 Limitations

The results drawn from this study should be approached with caution, given the limitations related to the generalizability of its results, data quality, and real-world applicability. Drawing exclusively from a single center's stroke registry restricts the wider relevance of our results. Moreover, the absence of vital data, such as imaging and laboratory results, may compromise the precision of our stroke mortality predictions.

The study's retrospective design could introduce biases. The reliance on historical data limits the model's capacity to capture the dynamic and changing treatment strategies, with the chosen one-year mortality prediction horizon not capturing the full spectrum of long-term patient outcomes. Challenges extend to the dataset's class imbalance and the complex nature of machine learning models, which could hinder model efficacy, and integration into clinical settings. Despite our efforts to address these limitations, residual biases and interpretability issues may remain.

The real-world value of machine learning models hinges on their deployment in clinical environments. However, clinical adoption faces obstacles, notably from the varying willingness of healthcare professionals to integrate AI-based tools into their practice. This hesitancy is compounded by the need for a nuanced understanding of ethical, legal, and potential bias implications. Effective human-AI interaction, ensuring clear and reciprocal comprehension between AI outputs and medical practitioners, is crucial for the successful integration of AI into healthcare protocols, thereby facilitating broader clinical acceptance.

Recognizing these limitations is critical for grasping both the capabilities and constraints of applying machine learning to improve healthcare outcomes. It highlights the imperative for ongoing research and refinement to address these challenges, ensuring machine learning's potential can be fully realized in enhancing patient care.

## 7 Conclusions

This study harnessed the potential of machine learning to craft a predictive model for stroke mortality, yielding notable accuracy and performance. Crucially, the model's incorporation of well recognized easily gathered predictors such as NIHSS at admission, age, hospital LOS, mode of arrival, HR, BP, and BMI – holds promising potential in personalizing stroke care. These insights provide avenues for early risk assessment, personalized clinical strategies, and improved patient and family expectations.

Nonetheless, limitations stemming from data quality and the absence of potentially significant variables resulted in moderate F1 score precision and recall. This highlights the crucial need to enhance data quality within the registry and broaden variable inclusivity.

With the recent popularization of personalized medicine, the study's outcomes underscore the need for prospective validation to affirm its applicability and reliability in real-world clinical settings. Successful implementation of these predictive tools could usher in an era of Artificial intelligence (AI) driven patient-centric care and more effective preventive approaches, thereby transforming the landscape of stroke management.

## Statement of ethics

The IRB at the Medical Research Center of Hamad Medical Corporation approved this project (MRC-01-22-594). The research was conducted in compliance with the ethical principles outlined in the Helsinki Declaration of 1964 and its subsequent modifications, as well as related ethical norms. The need for informed consent was waived by the IRB at the Medical Research Center of Hamad Medical Corporation.

## Funding sources

This study was not funded.

## Data availability statement

Data will be made available on request.

## CRediT authorship contribution statement

**Ahmad A. Abujaber:** Conceptualization, Data curation, Formal analysis, Writing – original draft, Writing – review & editing. **Ibrahem Albalkhi:** Conceptualization, Writing – original draft, Writing – review & editing, Data curation, Formal analysis. **Yahia Imam:** Conceptualization, Data curation, Writing – original draft, Writing – review & editing. **Abdulqadir Nashwan:** Data curation, Writing – original draft, Writing – review & editing. **Naveed Akhtar:** Data curation, Writing – original draft, Writing – review & editing. **Ibraheem M. Alkhawaldeh:** Conceptualization, Data curation, Formal analysis, Writing – original draft, Writing – review & editing.

## Declaration of competing interest

The authors declare that they have no known competing financial interests or personal relationships that could have appeared to influence the work reported in this paper.
